# Assessing the Diagnostic Accuracy of ChatGPT-4 in Identifying Diverse Skin Lesions Against Squamous and Basal Cell Carcinoma

**DOI:** 10.2196/67299

**Published:** 2025-03-21

**Authors:** Nitin Chetla, Matthew Chen, Joseph Chang, Aaron Smith, Tamer Rajai Hage, Romil Patel, Alana Gardner, Bridget Bryer

**Affiliations:** 1 University of Virginia School of Medicine, University of Virginia, 828 Cabell Avenue, Charlottesville, VA, 22903, United States, 1 571-581-0562; 2 Renaissance School of Medicine at Stony Brook University, Stony Brook, NY, United States; 3 University of Passau, Passau, Germany; 4 Virginia Tech, Blacksburg, VA, United States; 5 University at Albany, State University of New York, Albany, NY, United States; 6 Department of Dermatology, University of Virginia, Charlottesville, VA, United States

**Keywords:** chatbot, ChatGPT, ChatGPT-4, squamous cell carcinoma, basal cell carcinoma, skin cancer, skin cancer detection, dermatoscopic image analysis, skin lesion differentiation, dermatologist, machine learning, ML, artificial intelligence, AI, AI in dermatology, algorithm, model, analytics, diagnostic accuracy

## Abstract

Our study evaluates the diagnostic accuracy of ChatGPT-4o in classifying various skin lesions, highlighting its limitations in distinguishing squamous cell carcinoma from basal cell carcinoma using dermatoscopic images.

## Introduction

Squamous cell carcinoma (SCC) and basal cell carcinoma (BCC) are prevalent skin cancers that can cause significant local tissue damage and disfigurement as well as mortality in cases of aggressive SCCs [1,2]. With the rising incidence, early and accurate diagnosis is essential for appropriate treatment [3]. Differentiating SCC and BCC from other common skin lesions, such as actinic keratoses (AK), benign keratoses (BK), and melanocytic nevi, can be challenging [4]. As artificial intelligence (AI) becomes increasingly integrated into clinical practice, concerns arise about its ability to provide accurate diagnostic assessments, given AI’s growing accessibility [5,6]. We assessed the ability of ChatGPT to distinguish images of SCC and BCC from other lesions.

## Methods

OpenAI’s application programming interface was used to query ChatGPT-4 Omni (ChatGPT-4O) for assessing the performance in classifying 200 dermatoscopic images each of SCC, BCC, BK, melanocytic nevi, and 150 images of AK from the HAM10K database [7]. Images were verified using histopathology (>50%), follow-up examination, expert consensus, or in-vivo confocal microscopy. Two standardized prompts were used:

### Prompt 1

This is an image on the Step 1 examination, and the multiple-choice question is as follows: Based on the image, does the patient have (A) Nevus, (B) Actinic Keratosis (AK), (C) Benign Keratosis (BK), or (D) BCC, or (E) SCC. Only output (A), (B), (C), (D) or (E).

### Prompt 2

This is an image from a patient. Based on the image, does the patient have (A) Nevus, (B) AK, (C) BK, (D) BCC, or (E) SCC. Only output (A), (B), (C), or (D) or (E).

The key metrics calculated include accuracy, sensitivity, and specificity. Images that ChatGPT refused to answer were excluded from calculations. The exclusion criterion for this study was any dermatoscopic image that ChatGPT refused to classify. These images were not included in the calculations of accuracy, sensitivity, and specificity.

The study did not employ further prompt engineering to enhance ChatGPT’s performance because the goal was to evaluate its diagnostic accuracy using straightforward, unrefined prompts that reflect real-world scenarios. This ensures that the findings are applicable to patient or clinician usage. Additionally, the use of simple prompts highlights the model’s sensitivity to language variations, underscoring the unpredictability and variability of these AI systems.

## Results

For Prompt 1, ChatGPT classified nevi with an accuracy of 79.3% (95% CI 76.7%‐81.9%), sensitivity of 0.844, and specificity of 0.758. The accuracy for classifying BCC was 77.8% (95% CI 75.2%‐80.4%), with low sensitivity (0.081) and high specificity (0.959). The accuracy for classifying SCC was 66.1% (95% CI 52.8%‐59.2%), with sensitivity of 0.477 and specificity of 0.711 (Table 1).

In Prompt 2, SCC accuracy increased to 72.8% (95% CI: 70.0%‐75.6%) but sensitivity dropped to 0.245. Nevi accuracy slightly declined to 72.8%, while SCC specificity improved to 0.857 (Table 2).

**Table 1.** Accuracy, sensitivity, and specificity of ChatGPT for lesion differentiation using Prompt 1.

Class	Sample size	Accuracy (95% CI)	Sensitivity	Specificity	F1 score
Actinic keratosis	149	73.0% (70.2‐75.8)	0.356	0.802	0.294
Basal cell carcinoma	198	77.8% (75.2‐80.4)	0.081	0.959	0.132
Nevus	199	79.3% (76.7‐81.9)	0.844	0.758	0.649
Benign keratosis	200	74.4% (71.6‐77.2)	0.090	0.939	0.138
Squamous cell carcinoma	199	66.1% (52.8‐59.2)	0.477	0.711	0.373

**Table 2.** Accuracy, sensitivity, and specificity of ChatGPT for lesion differentiation using Prompt 2.

Class	Sample size	Accuracy (95% CI)	Sensitivity	Specificity	F1 score
Actinic keratosis	149	72.9% (70.1‐75.7)	0.423	0.774	0.329
Basal cell carcinoma	200	79.5% (76.9‐82.1)	0.07	0.987	0.125
Nevus	200	72.8% (70.0‐75.6)	0.89	0.664	0.58
Benign keratosis	200	73.7% (70.9‐76.5)	0.18	0.885	0.223
Squamous cell carcinoma	200	72.8% (70.0‐75.6)	0.245	0.857	0.275

## Discussion

ChatGPT-4o struggled to differentiate between SCC and BCC. Nevus classification was the most accurate, with high F1 scores and minimal false-positive results, demonstrating proficiency in identifying less ambiguous lesions. The model showed significant bias in SCC classification, frequently misclassifying SCC as BCC with a high rate of false-positive results. This aligns with previous research that observed SCC is often mistaken for BCC, particularly when features like pigmentation or rolled borders overlap [8]. ChatGPT’s performance worsened in Prompt 2, where SCC was frequently misclassified as AK. Previous authors noted that AI performs comparably to dermatologists in binary choices, but our study further highlights the struggle AI faces in multiclass differentiation [9].

Prompt 1 was designed to emulate a standardized examination scenario, leveraging ChatGPT’s ability to respond to structured, multiple-choice questions within a controlled academic framework. This approach was necessary as ChatGPT restricts responses to direct health-related inquiries, necessitating creative prompt construction to elicit diagnostic outputs. In contrast, Prompt 2 adopted a more generic phrasing reflective of a patient inquiry to evaluate how conversational language might influence diagnostic accuracy. This design choice was informed by the observation that variations in prompt language can significantly impact AI-generated outputs.

Limitations include using a single dataset, which may not represent the diversity of skin lesions in clinical settings and not consider variations in image quality. Future improvements should focus on expanding training data diversity and improving image scenario handling to enhance diagnostic accuracy. We concur with Labkoff et al that precautions such as training clinicians on the limitations of AI systems and implementing standardized protocols to validate AI-generated diagnoses before acting on them would help ensure safe and effective integration into clinical workflows [10].

## Abbreviations

AI: artificial intelligence
AK: actinic keratoses
BCC: basal cell carcinoma
BK: benign keratoses
SCC: squamous cell carcinoma
